# GeoIoU-SEA-YOLO: An Advanced Model for Detecting Unsafe Behaviors on Construction Sites

**DOI:** 10.3390/s25041238

**Published:** 2025-02-18

**Authors:** Xuejun Jia, Xiaoxiong Zhou, Zhihan Shi, Qi Xu, Guangming Zhang

**Affiliations:** 1 College of Electrical Engineering and Control Science, Nanjing Tech University, Nanjing 211816, China; jxj@njtech.edu.cn (X.J.); zhouxx@njtech.edu.cn (X.Z.); szh@njtech.edu.cn (Z.S.); xenon@njtech.edu.cn (Q.X.); 2 China Construction Second Engineering Bureau Co., Ltd., Beijing 100160, China

**Keywords:** unsafe behavior detection, GeoIoU, Structural-Enhanced Attention (SEA), YOLO, construction safety

## Abstract

Unsafe behaviors on construction sites are a major cause of accidents, highlighting the need for effective detection and prevention. Traditional methods like manual inspections and video surveillance often lack real-time performance and comprehensive coverage, making them insufficient for diverse and complex site environments. This paper introduces GeoIoU-SEA-YOLO, an enhanced object detection model integrating the Geometric Intersection over Union (GeoIoU) loss function and Structural-Enhanced Attention (SEA) mechanism to improve accuracy and real-time detection. GeoIoU enhances bounding box regression by considering geometric characteristics, excelling in the detection of small objects, occlusions, and multi-object interactions. SEA combines channel and multi-scale spatial attention, dynamically refining feature map weights to focus on critical features. Experiments show that GeoIoU-SEA-YOLO outperforms YOLOv3, YOLOv5s, YOLOv8s, and SSD, achieving high precision (mAP@0.5 = 0.930), recall, and small object detection in complex scenes, particularly for unsafe behaviors like missing safety helmets, vests, or smoking. Ablation studies confirm the independent and combined contributions of GeoIoU and SEA to performance gains, providing a reliable solution for intelligent safety management on construction sites.

## 1. Introduction

Safety management on construction sites has always been a critical research area in the construction industry. The complex and dynamic nature of construction site environments, combined with the unpredictability of worker behaviors and the heavy reliance on machinery, has led to frequent safety incidents, such as falls, electric shocks, and mechanical injuries [1,2]. Statistics indicate that the majority of construction accidents are directly related to unsafe worker behaviors, such as failing to wear personal protective equipment (PPE) properly, entering hazardous areas without authorization, or engaging in improper operations. These incidents not only endanger workers’ lives, but also result in significant economic losses and societal impacts.

Traditional methods for construction site safety management, including manual inspections and video surveillance, suffer from poor real-time performance, low efficiency, and limited coverage, making them inadequate to meet the growing safety management demands of modern construction sites [3,4]. To address these issues, researchers have begun leveraging computer vision technologies to identify and intervene in unsafe behaviors through automated monitoring systems. In particular, the rapid development of object detection technology has provided technical support for construction site safety management.

Object detection technology, which involves identifying and localizing objects of interest within images, has become a cornerstone for monitoring unsafe behaviors on construction sites. Early methods, such as Region-based Convolutional Neural Networks (R-CNN) [5] and single-stage detection models like SSD [6], achieved a balance between detection accuracy and speed. However, due to the complexity of construction environments, including the prevalence of small objects and partial occlusions, the performance of existing methods remains limited in handling these challenges. In recent years, the YOLO (You Only Look Once) series of algorithms have gained widespread application due to their efficiency and robustness, demonstrating exceptional performance in tasks such as safety helmet detection [7], reflective vest usage monitoring [8], and the identification of other unsafe behaviors on construction sites. Moreover, deep learning-based approaches, such as convolutional neural networks (CNNs), have been employed to enhance detection accuracy and real-time monitoring capabilities. Zhang developed a CNN-based framework capable of automatically analyzing surveillance footage to identify unsafe behaviors and provide real-time alerts [9]. Similarly, Yu introduced a skeleton-based motion analysis technique that effectively detects high-risk worker postures in real time [10].

In addition to object detection, pose estimation is another critical technology for detecting unsafe behaviors. By locating and analyzing the key skeletal points of workers, pose estimation methods can identify improper actions or abnormal postures during high-altitude operations. For example, studies have developed detection systems combining deep learning and pose analysis, capable of analyzing workers’ postures in real time and identifying potential hazardous behaviors [11,12]. A recent deep learning framework, DeepSafety, has been introduced to detect unsafe behaviors using both object detection and pose estimation. Liu and Ying proposed an approach that integrates YOLOv3 for worker detection with long short-term memory (LSTM) networks to analyze movement patterns and identify hazardous actions [13]. Despite its unique advantages in behavior analysis, pose estimation still faces challenges in scenarios involving multi-object detection and occlusions.

In summary, unsafe behavior monitoring systems based on object detection and pose estimation have proven to be effective tools for improving safety management on construction sites. Numerous studies in recent years have not only validated the effectiveness of these technologies, but also laid a solid foundation for the realization of real-time and efficient construction site safety monitoring systems [14,15]. In addition, behavior-based safety (BBS) approaches are increasingly being enhanced by artificial intelligence and computer vision technologies. Fang reviewed advancements in computer vision for BBS and highlighted the challenges associated with occlusions, varied lighting conditions, and viewpoint changes [16]. Furthermore, Huang examined the role of incentive structures in reducing unsafe behaviors, demonstrating that a combination of positive reinforcement and penalties can effectively promote safer workplace practices [17].

To address the diverse and complex requirements of unsafe behavior recognition in construction site environments, researchers have continually worked to improve object detection frameworks and their key components, aiming to enhance detection accuracy and real-time performance. However, challenges such as small objects, occlusions, and multi-object interactions remain significant in construction scenarios, limiting the applicability of the existing methods. To overcome these difficulties and meet growing demands, this paper proposes a construction site unsafe behavior detection model that integrates the Geometric IoU (GeoIoU) loss function with Structural-Enhanced Attention (SEA). Built on the YOLO framework, the model focuses on optimizing two critical aspects: bounding box regression and multi-scale feature extraction. The goal is to achieve efficient and accurate detection of various unsafe behaviors in complex construction environments.

This paper’s primary innovations and contributions are summarized as follows:

(**1**) **Proposal of an efficient object detection model for unsafe behavior detection on construction sites: GeoIoU-SEA-YOLO**

An efficient object detection model, GeoIoU-SEA-YOLO, is proposed based on the classical YOLO framework. By integrating the GeoIoU loss function and Structural-Enhanced Attention (SEA), the model achieves a balance between detection accuracy and real-time performance in complex construction environments. Compared with the existing methods, GeoIoU-SEA-YOLO demonstrates superior adaptability and robustness in handling small objects, partial occlusions, and multi-object scenarios.

(**2**) **Design and implementation of a novel GeoIoU loss function**

Unlike traditional IoU and its variants (GIoU, DIoU, CIoU), GeoIoU places greater emphasis on the geometric distribution relationships of bounding boxes, providing more stable and accurate gradient signals for bounding box regression. In experiments on unsafe behavior detection in construction sites, GeoIoU significantly improves the localization accuracy of the model.

(**3**) **Introduction of Structural-Enhanced Attention (SEA)**

The SEA mechanism integrates static/dynamic channel attention and multi-scale spatial attention through residual connections and a learnable fusion strategy, enhancing the model’s focus on critical features. Dual-Scale Attention addresses, to some extent, the insufficient feature representation caused by the diversity of and scale variations in targets in construction site scenarios.

(**4**) **Construction and public release of a multi-source synthetic dataset for unsafe behavior detection**

A multi-source synthetic dataset of unsafe behaviors on construction sites was constructed and thoroughly evaluated. Although comprehensive testing across multiple datasets and extreme environments was not performed, experimental results show that GeoIoU-SEA-YOLO outperforms various benchmark models in recognizing typical unsafe behaviors, such as detecting safety helmets, reflective vests, and certain violations. This evaluation demonstrates the model’s practical application potential in terms of both accuracy and inference speed.

## 2. Related Work

In recent years, object detection technology has been extensively studied, with the YOLO series of algorithms becoming a focal point of research due to their efficiency and robustness. Additionally, advancements in Intersection over Union (IoU) improvements and the introduction of attention mechanisms have further enhanced object detection performance. This section provides an overview of the related work, focusing on three key areas: major variants of YOLO, IoU improvements, and attention mechanisms.

### 2.1. Main Variants of YOLO

The YOLO series of algorithms have evolved rapidly since their inception, with key variants including Tiny-YOLO, Anchor-Free YOLO, and Transformer-YOLO, among others. Tiny-YOLO is a lightweight variant designed for embedded systems and real-time detection, capable of delivering satisfactory performance in low-resource environments, albeit at the cost of reduced detection accuracy [18]. Anchor-Free YOLO eliminates the traditional anchor box mechanism in favor of center point prediction, effectively reducing computational demands in complex scenarios and improving the detection of occluded objects [19]. Moreover, Transformer-YOLO leverages the global feature modeling capabilities of Transformers, demonstrating superior performance in unstructured data and long-range dependency scenarios [20].

Recently, road scene understanding datasets such as Rsud20k [21] have driven the development of object detection approaches tailored for autonomous driving. These datasets provide diverse and challenging real-world conditions, including occlusions, varying lighting conditions, and dynamic objects, which necessitate robust and efficient detection models. Several recent detection models, such as DETR [22] and DINO, have shown promising results by leveraging self-attention mechanisms to enhance long-range dependencies and improve detection performance in such complex scenarios. Additionally, the latest versions of YOLO, such as YOLOv7 and YOLOv8, have introduced optimizations in architectural design and training strategies, further improving efficiency and accuracy in real-time applications.

**Figure 1:**
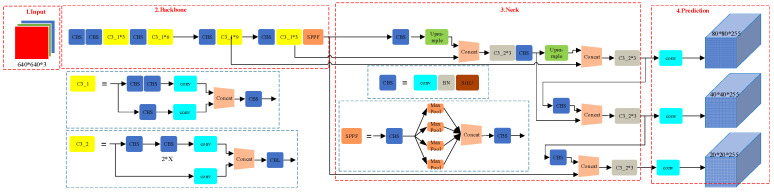
YOLOv5 network structure diagram.

Dense-YOLO is a variant tailored for dense object detection tasks, significantly enhancing detection performance in crowded scenes through improved feature fusion modules [21]. Meanwhile, YOLOx optimizes target matching through a dynamic label assignment mechanism and incorporates efficient loss functions, excelling in multi-object detection tasks [22]. Another notable variant is YOLOv5, an unofficial version of the YOLO series that has gained widespread application due to its outstanding performance and open-source nature. YOLOv5 introduces innovations in network architecture and training methods, achieving a better balance between accuracy, speed, and model size. It also supports various post-processing techniques, such as Non-Maximum Suppression (NMS) and Soft-NMS, effectively improving detection precision. Figure 1 illustrates the network structure of YOLOv5, highlighting its optimizations, which make it a pivotal tool in modern object detection. The diagram depicts the flow of data through the network, starting from the input layer (on the left) and progressing through various components. These include the convolutional layers, which extract features from the input image, as well as the fully connected layers responsible for detecting objects. The output layer (on the far right) produces the final object detection results. Notably, Figure 1 also emphasizes the introduction of key architectural components, such as the modified residual blocks and advanced up-sampling strategies, which contribute to YOLOv5’s superior performance, especially in real-time detection scenarios.

### 2.2. Advances in IoU-Based Research

Intersection over Union (IoU) is a crucial metric in object detection for measuring the overlap between predicted and ground truth bounding boxes. However, IoU performs poorly for objects with significant aspect ratio differences. To address this limitation, several enhanced variants of IoU have been proposed. DIoU incorporates the distance between the center points of bounding boxes, optimizing localization accuracy and significantly accelerating model convergence [23]. CIoU builds on DIoU by additionally considering the similarities in the aspect ratios of bounding boxes, improving performance in small object detection tasks [24]. SIoU targets issues in Non-Maximum Suppression for complex scenarios, optimizing the allocation strategy of bounding boxes and substantially reducing false positive and false negative rates [25]. Adaptive IoU further addresses challenges in multi-scale object detection by dynamically adjusting the weight of the IoU loss function. This approach is particularly effective for detection tasks in complex backgrounds [26]. These advancements have significantly enhanced the robustness of object detection models in complex environments.

### 2.3. Applications of Attention Mechanisms

In recent years, attention mechanisms have been extensively applied in object detection tasks, significantly enhancing the representation of critical features through dynamic weight allocation. The Squeeze-and-Excitation (SE) module leverages global pooling to compute feature weights, amplifying the representation of essential channels and delivering outstanding performance in multi-object detection [27]. The Convolutional Block Attention Module (CBAM) combines channel and spatial attention, achieving notable improvements in precision in the detection of small objects [28]. Triplet Attention introduces a contextual enhancement mechanism that extends interactions across both channel and spatial dimensions, enabling the precise detection of occluded objects [29]. Transformer Attention, by employing a multi-head attention mechanism, captures global features with remarkable effectiveness, particularly in scenarios involving long-range dependencies [20]. Lightweight modules such as Efficient Channel Attention (ECA) further enhance feature extraction capabilities while maintaining minimal computational cost, making them exceptionally suitable for real-time detection tasks [30]. These advancements underscore the critical role of attention mechanisms in improving the accuracy, robustness, and efficiency of modern object detection systems.

## 3. Method

### 3.1. Overall Architecture

**Figure 2:**
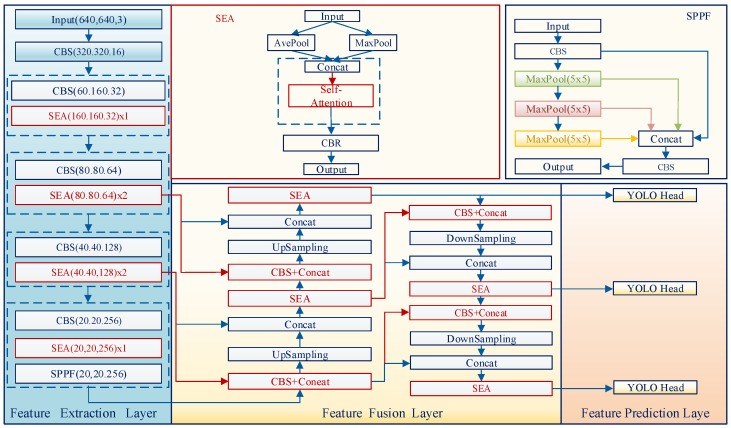
The architecture of the SEA-YOLO network.

To comprehensively improve the detection accuracy and real-time performance of unsafe behaviors on construction sites, this paper proposes the GeoIoU-SEA-YOLO model. The architecture of this model is designed to enhance both feature extraction and bounding box regression, addressing challenges such as small object detection, occlusions, and multi-object detection in complex construction site environments. The network flow of the SEA-YOLO model is shown in Figure 2.

The model follows a multi-stage workflow, starting from the input image and ending with the final detection results. The first step in the process involves feature extraction, where the input image (size 640 × 640) passes through a series of CBS layers (Convolution, Batch Normalization, and Activation). These layers extract low-level features, such as edges and textures, from the input image. Next, the extracted features undergo feature fusion, where information from multiple feature maps is combined. This process is enhanced by the SEA (Structural-Enhanced Attention) module, which focuses on the most critical regions of the feature map, allowing the model to adaptively allocate attention based on spatial and channel-specific characteristics. The SEA module enhances the model’s ability to capture both local and global dependencies, which is particularly important in scenarios where objects may be partially occluded or of varying sizes. Following feature fusion, the model proceeds with down-sampling and up-sampling operations, which help maintain the balance between computational efficiency and the model’s ability to capture fine-grained features. The up-sampled features are then passed to the next stage, where further fusion and attention mechanisms are applied. After the final feature fusion, the processed features are passed to the YOLO head, which predicts the bounding boxes and class labels for the detected objects. The output layer then provides the final detection results, which include the predicted bounding boxes and associated confidence scores.

Throughout the process, the GeoIoU loss function is applied during training to refine the bounding box predictions. The GeoIoU loss function improves the localization accuracy by considering geometric relationships such as the Manhattan distance, Euclidean distance, and aspect ratio similarity between the predicted and ground truth bounding boxes. This approach ensures more stable and accurate gradient signals during the optimization process.

### 3.2. GeoIoU Loss Function

In object detection tasks, the design of the loss function is critical to the performance of the model. The traditional Intersection over Union (IoU), widely used in various object detection models as a metric to measure the overlap between predicted and ground truth bounding boxes, has proven effective. However, for the specific application scenario of detecting unsafe behaviors on construction sites, the traditional IoU exhibits significant limitations when dealing with small objects, partial occlusions, and multi-object interactions in complex environments. To address these challenges, this paper proposes a novel GeoIoU loss function, which aims to enhance the precision and stability of bounding box regression by incorporating geometric characteristics.

#### 3.2.1. Motivation for the Design of GeoIoU

The traditional IoU loss function primarily relies on the overlap between predicted and ground truth bounding boxes to guide model optimization. However, in the context of detecting unsafe behaviors on construction sites, several key challenges arise:

1. **Difficulty in Detecting Small Objects**: Many unsafe behaviors on construction sites involve detecting small objects, such as safety helmets and reflective vests. In these cases, the traditional IoU struggles because the small size of the bounding boxes amplifies the impact of localization errors, causing significant fluctuations in the IoU values. This, in turn, affects the stability of model training and slows convergence;
2. **Partial Occlusion and Multi-Object Interaction**: The complexity of construction site environments often results in partial occlusions or overlaps between objects. In such scenarios, the intersection area between bounding boxes may decrease significantly, leading to reduced IoU values. This can misguide the optimization process, making it difficult for the predicted bounding boxes to accurately cover the true objects;
3. **Neglect of Geometric Relationships**: Traditional IoU focuses solely on the degree of overlap and neglects the geometric distribution between bounding boxes. For example, two bounding boxes may have similar IoU values but differ significantly in relative position or shape, directly impacting detection performance.

To address these issues, GeoIoU introduces the geometric characteristics of bounding boxes, comprehensively considering the distance between their centers and their shape similarity. By providing more precise and stable loss signals, GeoIoU aims to enhance the model’s detection performance in complex construction site environments.

#### 3.2.2. Mathematical Definition of GeoIoU

The GeoIoU loss function extends the traditional IoU by incorporating the geometric distribution information of bounding boxes to provide richer optimization signals. Its mathematical definition is as follows:

Let the predicted bounding box be $B_{p}=(x_{p},y_{p},w_{p},h_{p})$ and the ground truth bounding box be $B_{g}=(x_{g},y_{g},w_{g},h_{g})$, where (x,y) represents the center coordinates of the bounding box, and w and h represent its width and height, respectively. First, the traditional IoU is computed as follows:

(1) $$IoU=\frac{Area(B_{p}\cap B_{g})}{Area(B_{p}\cup B_{g})}$$

Next, we introduce the Manhattan distance $d_{x}+d_{y}$ between the center points of the bounding boxes, the Euclidean center distance center_dist, and the aspect ratio similarity aspect_diff:

(2) $$\begin{array}{c} d_{x}=|x_{p}-x_{g}|\\ d_{y}=|y_{p}-y_{g}|\\ center\_dist=\sqrt{d_{x}^{2}+d_{y}^{2}}\\ aspect\_ratio1=\frac{w_{p}}{h_{p}+\epsilon }\\ aspect\_ratio2=\frac{w_{g}}{h_{g}+\epsilon }\\ aspect\_diff=(aspect\_ratio1-aspect\_ratio2{)}^{2} \end{array}$$

The definition of *GeoIoU* is as follows:

(3) $$GeoIoU=IoU-\frac{d_{x}+d_{y}+\beta \cdot center\_dist+\gamma_{aspect}\cdot aspect\_diff}{c^{2}+\epsilon }$$

where

β and $\gamma_{aspect}$ are weight parameters used to balance the influence of center distance and shape difference.

$c^{2}=cw^{2}+ch^{2}$ is a normalization factor, where cw and ch are the average widths and heights of the predicted and ground truth bounding boxes, respectively.

ϵ is a small constant introduced to prevent division by zero.

In this formula,

$d_{x}+d_{y}$ is the Manhattan distance between the center points of the predicted and ground truth bounding boxes, reflecting the degree of horizontal and vertical offset.

center_dist is the Euclidean distance between the center points of the predicted and ground truth bounding boxes, providing a more precise quantification of spatial displacement.

aspect_diff represents the squared difference in aspect ratios between the bounding boxes, measuring the shape discrepancy between the predicted and ground truth bounding boxes.

**Figure 3:**
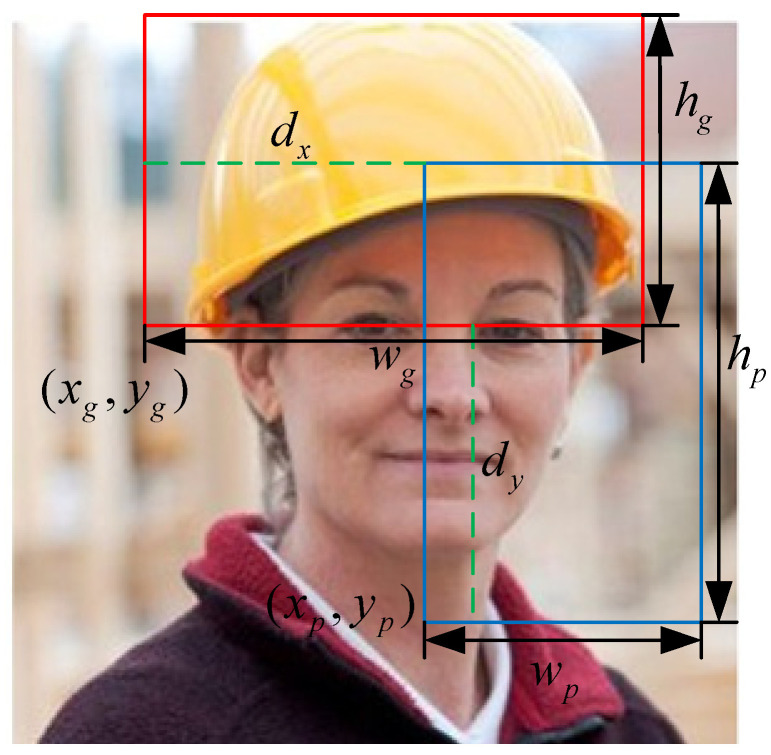
Schematic diagram of the GeoIoU structure.

A normalization factor $c^{2}$ is introduced to ensure that the geometric distances and shape differences have consistent impacts across varying scales. By incorporating geometric distances $d_{x}+d_{y}$, center distances center_dist, and shape similarity aspect_diff, GeoIoU extends beyond overlap-based metrics to include spatial relationships and shape consistency in the loss calculation. This design enables GeoIoU to provide more stable and accurate gradient signals during bounding box regression, significantly improving localization accuracy, particularly for small objects and in complex scenarios. The components of the formula are illustrated in Figure 3.

#### 3.2.3. Comparison of GeoIoU with Other IoU Variants

In recent years, with the advancement of object detection technologies, several IoU variants have been proposed to address the limitations of traditional IoU in specific application scenarios. Compared to GIoU, DIoU, and CIoU, GeoIoU offers the following advantages:

1. **Comprehensive Geometric Relationships**: GeoIoU not only considers the overlap of bounding boxes, but also incorporates center distance and shape similarity, providing more comprehensive optimization signals. This enables GeoIoU to better guide model optimization for object localization, especially in scenarios involving small objects and partial occlusions;
2. **Stable Gradient Signals**: By integrating geometric features, GeoIoU provides more stable and accurate gradient signals during bounding box regression, facilitating faster model convergence and precise localization;
3. **Adaptation to Complex Scenarios**: GeoIoU is designed with the complexity and diversity of construction site environments in mind. It demonstrates greater robustness and adaptability in handling multi-object interactions, partial occlusions, and small object detection.

In addition to detecting unsafe behaviors on construction sites, GeoIoU is applicable to other scenarios requiring high-precision bounding box regression, such as small object detection, dense object detection, and multi-object interaction detection in complex scenarios.

In summary, GeoIoU enhances the precision and stability of bounding box regression by integrating geometric features, building on the foundation of traditional IoU and its variants. Notably, in the challenging application of detecting unsafe behaviors on construction sites, GeoIoU demonstrates significant advantages, effectively addressing challenges in small object detection and localization in complex scenarios.

### 3.3. Structural-Enhanced Attention (SEA)

To further enhance the feature representation capabilities of the model in complex construction site environments, this paper proposes the Structural-Enhanced Attention (SEA) mechanism. By combining channel attention, self-attention, and spatial attention, the SEA mechanism dynamically adjusts the importance of different regions in the feature maps, thereby strengthening the model’s focus on critical features. The following sections provide a detailed introduction to the design principles, modular composition, and workflow of SEA.

#### 3.3.1. Design Philosophy of SEA

In the task of detecting unsafe behaviors on construction sites, targets exhibit diverse characteristics and are often surrounded by complex backgrounds. This poses challenges in feature extraction and target recognition. Traditional attention mechanisms improve feature representation but still face limitations, particularly in multi-scale feature fusion and selection under complex conditions. To address these, SEA introduces the following optimizations:

1. Channel Attention with Dynamic Weight Generation: SEA begins by capturing channel-wise information at different scales through a combination of global average pooling and max pooling. This allows the model to consider both coarse and fine-grained features. A 1D convolution is then used instead of a fully connected layer to model local channel relationships more effectively. This convolution-based approach provides more efficient learning of inter-channel dependencies. Additionally, SEA introduces dynamic weight generation through learnable fusion parameters α, which balance static and dynamic components, allowing for flexible and adaptive channel attention;
2. Integration of Self-Attention Mechanism: The self-attention module in SEA captures global dependencies across the entire feature map by considering relationships between all positions. This is particularly useful in scenarios where partial occlusion or complex backgrounds occur. Instead of relying only on localized features, the self-attention mechanism enables the model to focus on crucial areas by considering the full context of the image, helping to better recognize occluded or hidden targets;
3. Multi-Scale Spatial Attention: SEA employs multi-scale spatial attention using depth-wise separable convolutions to extract spatial features at different scales. This approach enables the model to detect objects of varying sizes by capturing features at multiple resolutions. Unlike single-scale mechanisms, this multi-scale method enhances the model’s robustness in detecting both small and large objects in diverse settings;
4. Residual Bottleneck Module: To ensure effective information flow and prevent gradient vanishing, SEA integrates a **residual bottleneck module**. This module introduces residual connections that help maintain important features throughout the network, ensuring efficient backpropagation and better overall model performance, especially in deeper architectures.

#### 3.3.2. Components of SEA

The SEA mechanism is primarily composed of the following modules:

1. Channel Attention Module

The channel attention module is designed to dynamically adjust the weights of each channel to emphasize critical features. The specific steps are as follows:

Pooling Operations:

(4) $$F_{avg}=\mathrm{AdaptiveAvgPool}2d\left(x\right)\left[B,C,1,1\right]\;F_{max}=\mathrm{AdaptiveMaxPool}2d(x)[B,C,1,1]$$

Here, x is the input feature map, B represents the batch size, and C denotes the number of channels.

1D Convolution and Fusion:

(5) $$\begin{array}{c} F_{cat}=\mathrm{Concat}(F_{avg},F_{max})[B,C,2]\\ F_{conv}=\mathrm{Conv}1d(F_{cat})[B,1,C]\\ F_{static}=\sigma (\mathrm{BN}1D({Conv1d}_{static}(F_{conv})))[B,C,1,1]\\ F_{dynamic}=\sigma (\mathrm{BN}1D({Conv1d}_{dynamic}(F_{conv})))[B,C,1,1]\\ F_{final}=\alpha \cdot F_{static}+(1-\alpha )\cdot F_{dynamic}[B,C,1,1] \end{array}$$

Here, α is a learnable fusion parameter and σ represents the Sigmoid activation function.

Feature Re-scaling:

(6) $$x_{channel}=x⊗F_{final}\left[B,C,H,W\right]$$

Here, ⊗ denotes element-wise multiplication.

2. Self-Attention Module

The self-attention module enhances feature representation by capturing global dependencies through the computation of relationships between all positions in the feature map. The specific steps are outlined below:

**Feature Transformation**

(7) $$Q=\theta \left(x\right)\left[B,C^{\prime },N\right]$$

(8) $$K=\phi \left(x\right)\left[B,C^{\prime },N\right]$$

(9) $$V=g(x)[B,C^{\prime },N]$$

Here, C′=C/8, N=H×W, and θ, ϕ, and g represent convolution operations.

Attention Weight Calculation:

(10) $$f=Softmax\left(\frac{Q\cdot K^{T}}{\sqrt{C^{\prime }}}\right)[B,N,N]$$

Feature Aggregation and Output:

(11) $$\begin{array}{c} y=f\cdot V[B,N,C^{\prime }]\\ y=y^{T}[B,C^{\prime },N]\\ y=\mathrm{Reshape}(y,[B,C^{\prime },H,W])\\ y=\mathrm{Conv}2d(y)[B,C,H,W]\\ \mathrm{out}=\gamma \cdot y+x[B,C,H,W] \end{array}$$

Here, γ is a learnable parameter.

3. Spatial Attention Module

The spatial attention module leverages multi-scale depth-wise separable convolutions to dynamically adjust the importance of various spatial positions in the feature map. The specific steps are outlined below:

Pooling Operations:

(12) $$F_{avg\_spatial}=Mean\left(x,dim=1,\mathrm{keepdim}=\mathrm{True}\right)\left[B,1,H,W\right]\;F_{max\_spatial}=Max(x,dim=1,\mathrm{keepdim}=\mathrm{True})[B,1,H,W]$$

Multi-Scale Convolution and Fusion:

(13) $$\begin{array}{c} F_{concat}=\mathrm{Concat}(F_{avg\_spatial},F_{max\_spatial})[B,2,H,W]\\ F_{spatial1}={\mathrm{DepthwiseSeparableConv}}_{3}(F_{concat})[B,1,H,W]\\ F_{spatial2}={\mathrm{DepthwiseSeparableConv}}_{5}(F_{concat})[B,1,H,W]\\ F_{spatial3}={\mathrm{DepthwiseSeparableConv}}_{7}(F_{concat})[B,1,H,W]\\ F_{spatial}=F_{spatial1}+F_{spatial2}+F_{spatial3}[B,1,H,W]\\ F_{spatial}=\sigma (F_{spatial})[B,1,H,W] \end{array}$$

Feature Re-scaling:

(14) $$x_{spatial}=x⊗F_{spatial}$$

4. **Residual Bottleneck**

The residual bottleneck module introduces residual connections to maintain the flow of information and prevent gradient vanishing, thereby further optimizing feature representation. The specific steps are as follows:

**Feature Transformation:**

(15) $$\begin{array}{c} F_{\mathrm{bottleneck}}=\mathrm{ReLU}\left(BN\left(Conv1\left(x\right)\right)\right)\left[B,C^{\prime },H,W\right]\\ F_{\mathrm{bottleneck}}=\mathrm{ReLU}\left(BN\left(Conv2\left(F_{\mathrm{bottleneck}}\right)\right)\right)\left[B,C,H,W\right] \end{array}$$

Residual Connection:

(16) $$out=F_{\mathrm{bottleneck}}+x\left[B,C,H,W\right]\;out=ReLU\left(out\right)\left[B,C,H,W\right]$$

**Figure 4:**
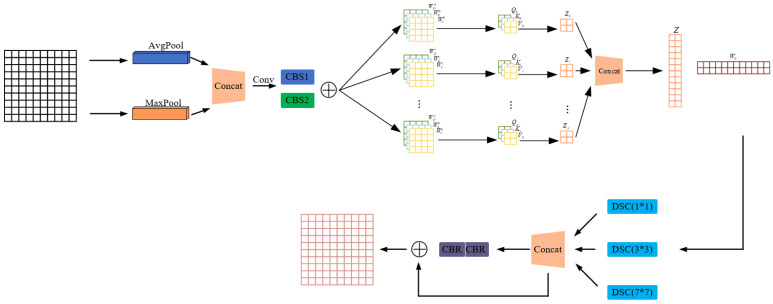
Workflow of the Structural-Enhanced Attention (SEA) mechanism.

The workflow of the SEA mechanism is shown in Figure 4.

## 4. Experiments

### 4.1. Experimental Environment and Datasets

All experiments and tests in this study were conducted under a consistent platform environment and training strategy, with the specific hardware and software configurations outlined in Table 1.

The datasets used are classified into three primary categories: safety helmets, reflective vests, and cigarettes. However, four detection labels are defined: safety helmets, humans, reflective vests, and smoking. The “human” label is included in all three datasets, and since there is already sufficient training data for this category, no additional datasets were incorporated.

**Figure 5:**
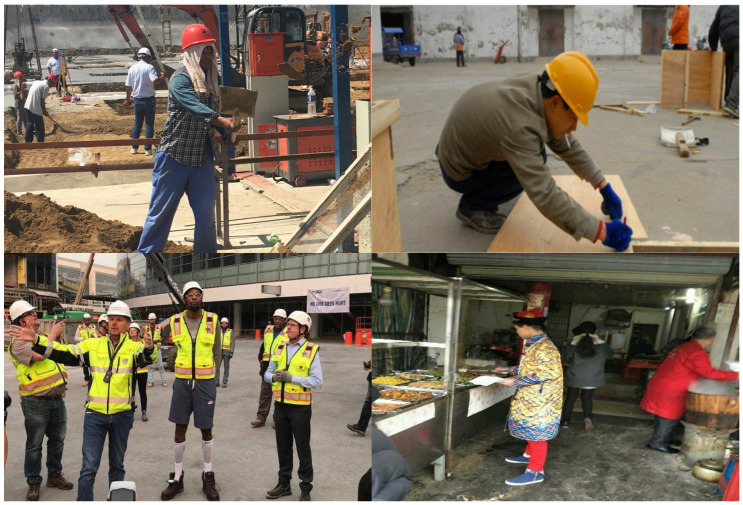
Example of datasets.

The complete dataset comprises 19,961 images. To evaluate the model’s generalization performance, the dataset is divided into a training set containing 14,835 images and a test set containing 5126 images. While most data originate from publicly available datasets, considering the focus on detecting unsafe behaviors on construction sites, additional datasets collected by our team from actual construction sites were included. Examples of typical images in this dataset are presented in Figure 5.

### 4.2. Comparison with Mainstream Methods

**Figure 6:**
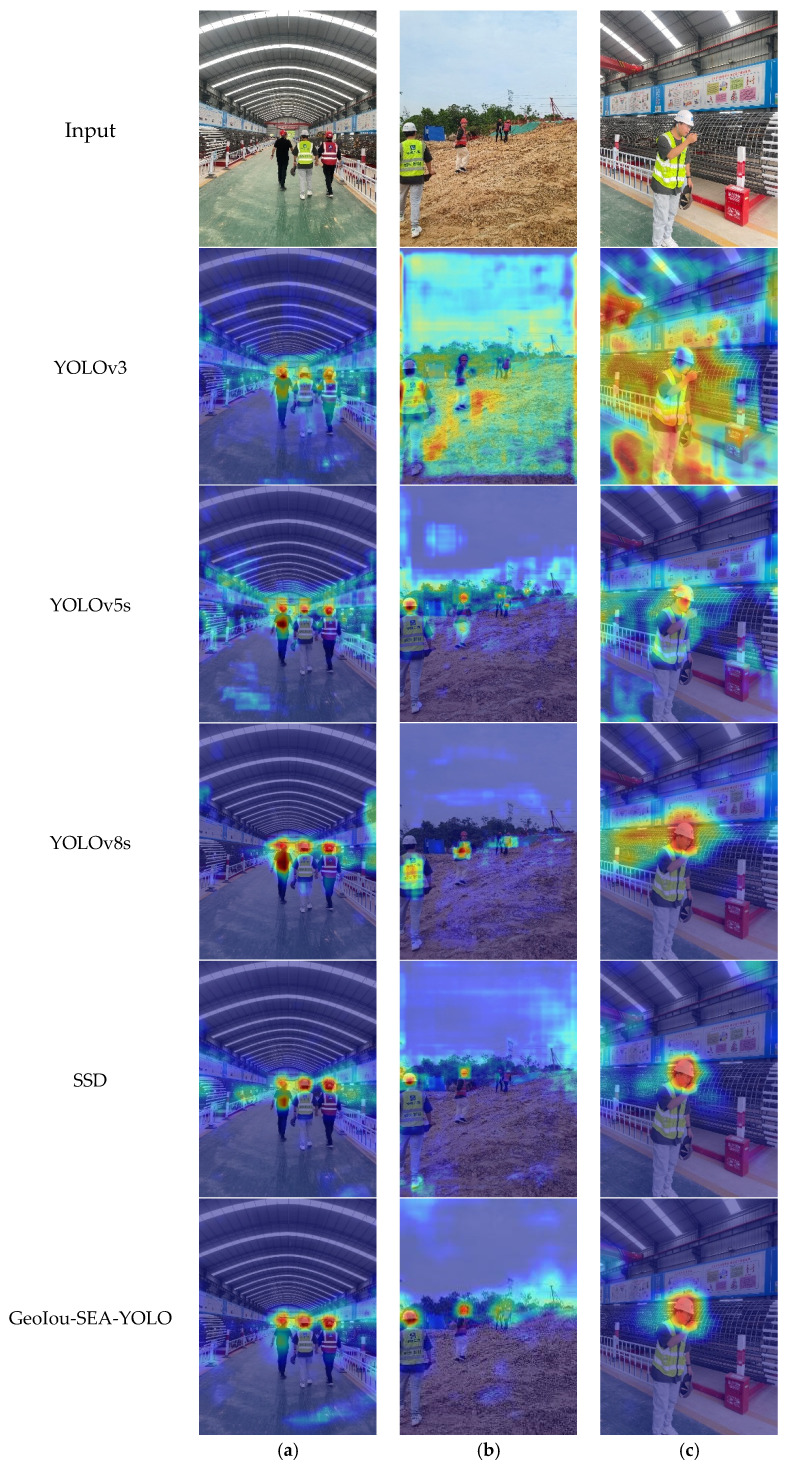
Comparison of Grad-CAM visualizations across different mainstream algorithms. (**a**–**c**) represent different picture examples.

To intuitively showcase the effectiveness of our improved model, we employed the Gradient Weighted Class Activation Mapping (Grad-CAM) method to visualize the key focus areas of the model. Comparisons were made with other mainstream detection methods, including YOLOv3, YOLOv5s, YOLOv8s, and SSD. This visualization highlights the attention regions of different models when processing the same image, shown as Figure 6.

The experimental results demonstrate that our proposed method achieves a more precise focus on target objects compared to other mainstream algorithms. This advantage is particularly notable in the detection of small objects, such as safety helmets, reflective vests, and cigarettes, where our model exhibits stronger robustness. Additionally, in complex backgrounds, such as construction site scenarios with numerous distractions and challenging environmental factors, our model consistently maintains high detection accuracy. Grad-CAM visualizations further validate that our model not only enhances detection accuracy, but also has an improved ability to focus on the fine details of target objects. These results confirm the improved method’s adaptability and reliability in complex environments, making it highly effective for practical applications.

Table 2 compares the predictive performance of the proposed GeoIoU-SEA-YOLO method with several mainstream algorithms, including YOLOv3, YOLOv5s, YOLOv8s, and SSD, on the specified dataset. The evaluation metrics include precision, recall, and mAP@0.5. The results demonstrate that GeoIoU-SEA-YOLO outperforms the other models across all metrics. In particular, the model achieves an AP of 0.957 for the **Smoking** target, highlighting its robustness in detecting complex targets such as small or occluded objects. Moreover, GeoIoU-SEA-YOLO achieves an mAP@0.5 of 0.930, significantly surpassing YOLOv3, YOLOv5s, YOLOv8s, and SSD, reflecting its superior overall detection performance. With a precision of 0.870 and a recall of 0.800, GeoIoU-SEA-YOLO demonstrates a strong balance between high detection accuracy and good recall capability. Although YOLOv8s is close in terms of AP and mAP@0.5, it lags behind GeoIoU-SEA-YOLO in detecting complex targets. SSD and YOLOv3 show comparatively lower performances, underscoring their limitations in handling challenging detection scenarios.

These findings affirm that GeoIoU-SEA-YOLO possesses significant advantages in object detection tasks, particularly in scenarios requiring high precision and robustness in complex environments.

From the above results, it can be seen that the model proposed in this paper can effectively improve the accuracy of detection on construction sites. However, in the actual construction site, the monitoring system needs to process a large amount of video data in real time. Therefore, the computational complexity and operation efficiency of the model are particularly critical. Table 3 lists the relevant parameters and data comparisons.

As can be seen from the above table, the GeoIoU-SEA-YOLO model is between YOLOv8s and SSD in terms of the number of parameters and FLOPs, and the inference speed is slightly lower than YOLOv8s, but significantly higher than Faster R-CNN. This shows that GeoIoU-SEA-YOLO optimizes computational complexity and operating efficiency while maintaining high accuracy, and is suitable for application scenarios that require real-time processing.

In the GeoIoU-SEA-YOLO model, we introduced the GeoIoU loss function and the Structural-Enhanced Attention (SEA) mechanism to improve the detection accuracy of the model in complex environments, especially when dealing with challenges such as small objects, occlusions, and multi-object interactions. Although these enhancements improve the accuracy of the model, they also increase the number of model parameters to a certain extent, and lead to a longer inference time. The purpose of introducing the GeoIoU loss function is to enhance the model’s positioning ability in small object detection, especially in complex scenes. GeoIoU provides a more stable and accurate gradient signal by considering the geometric relationship of the bounding box (such as center distance, Euclidean distance, and aspect ratio similarity), thereby improving the detection performance of the model. At the same time, the SEA mechanism further improves the model’s attention to key areas by dynamically adjusting the attention of channels and spaces in the feature map. Especially when the complex background or the target is partially occluded, SEA can effectively improve the detection accuracy.

Although the introduction of these technologies has increased the computational complexity of the model, the experimental results show that the GeoIoU-SEA-YOLO model has achieved a good balance between inference speed and accuracy. We verified in the experiment that the model can run at a speed of 28.6 frames per second, which fully meets the requirements of real-time monitoring. In addition, in the future, the model can be further optimized through model pruning, quantization, and other technologies to reduce the consumption of computing resources, so as to better meet the needs of actual deployment.

### 4.3. Ablation Study

A series of ablation experiments were designed to comprehensively evaluate the contributions of individual components in the proposed GeoIoU-SEA-YOLO model. By systematically removing or substituting key modules, the experiments quantify the impact of each component on the model’s overall performance. Furthermore, the independent contributions of the GeoIoU loss function and the SEA module were analyzed, providing further evidence of their effectiveness and the value they add to the model.

#### Independent Impact of GeoIoU and SEA

To investigate the independent contributions of the GeoIoU loss function and the SEA mechanism to the model’s performance, two experiments were designed:

(a) **Introducing GeoIoU Loss Function Only:**

This experiment keeps the SEA module unchanged while replacing the traditional IoU loss function with the GeoIoU loss function. The comparison of performance before and after the introduction of GeoIoU quantifies its impact on bounding box regression.

(b) **Introducing SEA Mechanism Only:**

This experiment retains the traditional IoU loss function while incorporating the SEA mechanism into the YOLO framework. Through a comparison with the baseline model that excludes SEA, the experiment evaluates SEA’s contribution to feature extraction and attention allocation.

The results in Table 4 demonstrate the significant contributions of the GeoIoU loss function and the SEA mechanism to model performance. The baseline YOLOv5s model achieved an mAP@0.5 of 0.822. When the GeoIoU loss function was incorporated, the mAP@0.5 improved to 0.829, highlighting its enhancement of precision. Introducing the SEA module further increased the mAP@0.5 to 0.855, emphasizing its role in improving recall. When both components were integrated into the GeoIoU-SEA-YOLO model, the mAP@0.5 reached 0.870, with a precision of 0.800 and a recall of 0.930, demonstrating the best overall performance. These results confirm the synergistic effect of the two modules in enhancing the model’s capabilities.

**Figure 7:**
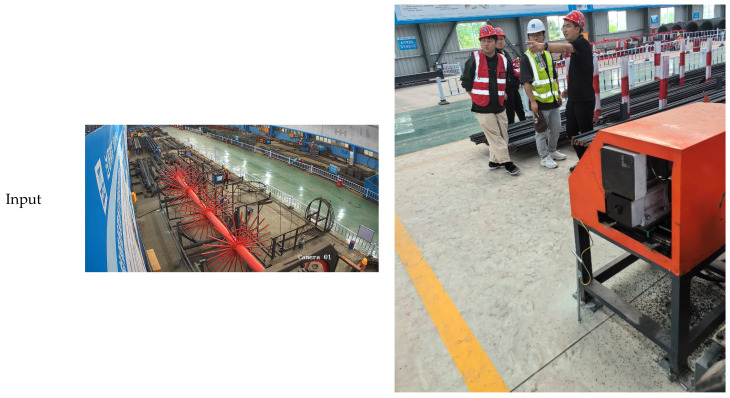

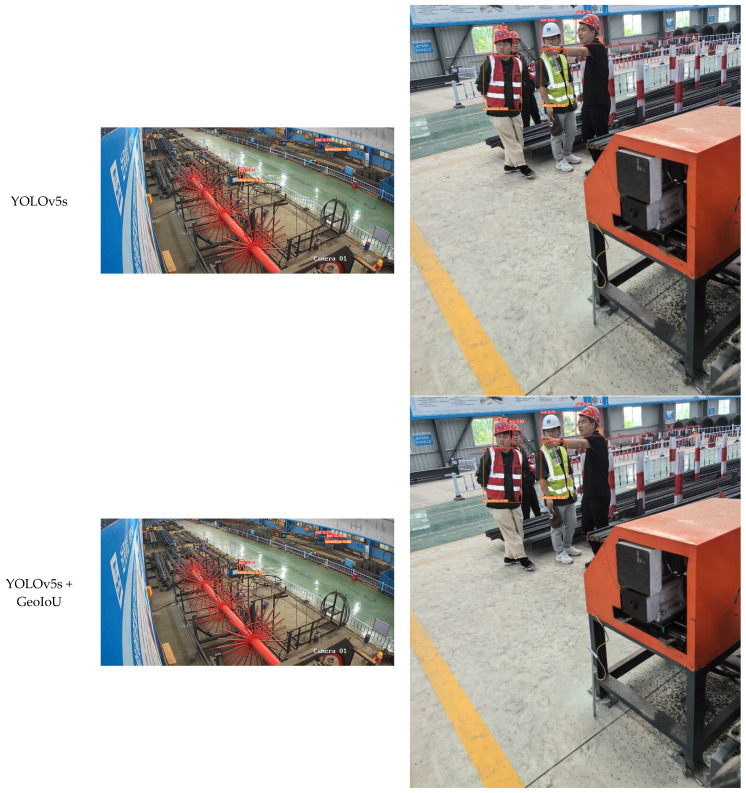

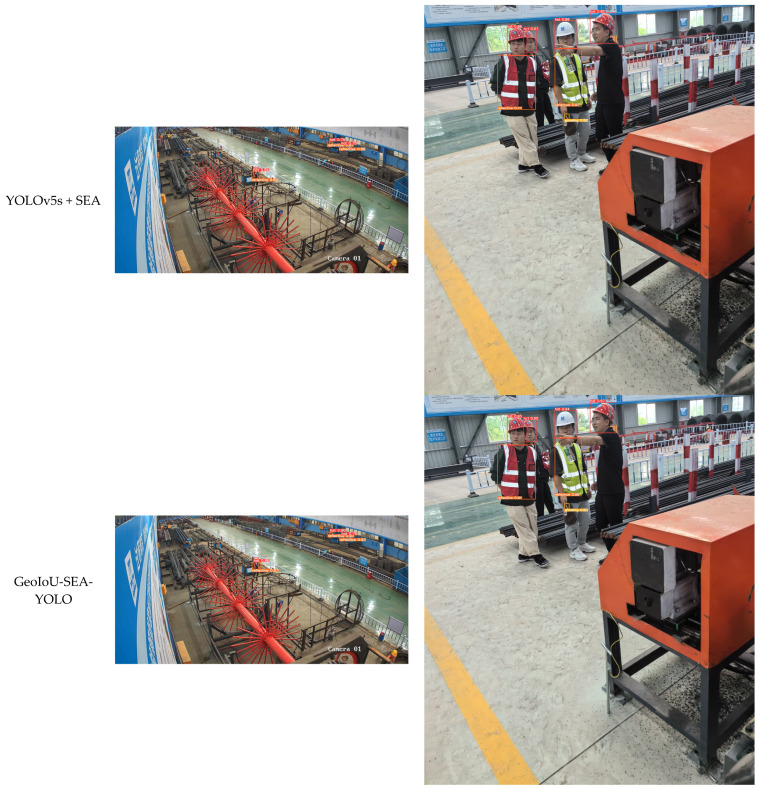
Comparison of detection results in ablation experiments.

Figure 7 provides a comparison of the detection results from the ablation experiments. The baseline YOLOv5s model detected most targets, but exhibited limitations in certain scenarios. For instance, in the first example, the baseline model failed to detect a small target due to its size. In the second example, a small target (cigarette) was not recognized, and an occluded safety helmet was missed. Although the model correctly identified larger targets, its confidence scores were relatively low.

Replacing the IoU function with GeoIoU significantly improved confidence scores, but small object detection challenges remained. Introducing the SEA mechanism enabled the model to accurately detect small targets, even those occupying minimal pixel areas, while also markedly improving the confidence levels for all detected objects. These results validate the effectiveness of the SEA module and its ability to enhance detection performance in complex scenarios.

**Figure 8:**
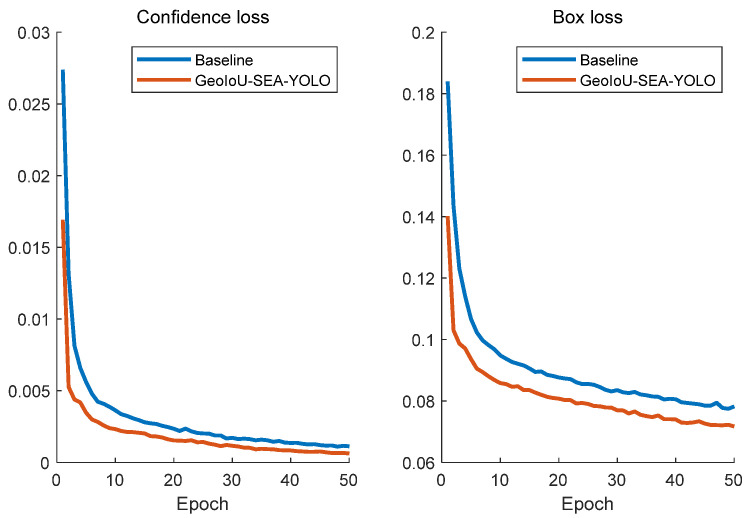
Comparison of loss function graph with baseline method.

Figure 8 illustrates the comparison of confidence loss and classification loss between the proposed GeoIoU-SEA-YOLO method and the baseline model. The results show that GeoIoU-SEA-YOLO consistently achieves lower loss values throughout the training process. In particular, the proposed method converges faster during the early stages and maintains more stable loss values in the later stages, with the performance gap persisting across the entire training duration.

This demonstrates the advantages of GeoIoU-SEA-YOLO in terms of optimization strategies, model architecture, and loss function design. These enhancements enable GeoIoU-SEA-YOLO to improve model performance effectively, potentially leading to higher detection accuracy and improved regression results. Consequently, GeoIoU-SEA-YOLO provides a more reliable and robust solution for object detection tasks, particularly in scenarios that demand high precision and reliability.

Although the GeoIoU-SEA-YOLO model has achieved good performance in the unsafe behavior detection task, there are still some limitations. First, the model is trained based on a specific construction site dataset, which is not yet publicly available, so the generalization ability of the model may be limited. Especially when applied to other different scenarios or datasets, the performance may be reduced. Second, although the introduction of the GeoIoU loss function and the SEA mechanism helps to improve detection accuracy, it also leads to an increase in the computational complexity and number of parameters of the model, which may affect the deployment and operation of the model on devices with limited computing resources. Nevertheless, the model can still maintain a high inference speed in real-time processing and is suitable for most real-time monitoring needs. Finally, although the model performs well in complex background and small object detection, the detection performance may be reduced when dealing with extremely complex environments (such as scenarios with multiple targets densely appearing or severely occluded). Therefore, future research can explore model optimization, dataset diversification, and customized adjustments in specific scenarios to further improve the model’s performance in different environments.

## 5. Conclusions

This paper proposes GeoIoU-SEA-YOLO, an object detection model that integrates the Geometric IoU (GeoIoU) loss function and the Structural-Enhanced Attention (SEA) mechanism, designed for detecting unsafe behaviors in complex construction site scenarios. By optimizing the geometric distribution characteristics of bounding box regression and enhancing the model’s ability to extract critical features, this model effectively addresses challenges such as small object detection, target occlusion, and multi-object interactions commonly encountered in construction environments.

The experimental results demonstrate that GeoIoU-SEA-YOLO outperforms mainstream detection methods, including YOLOv3, YOLOv5s, YOLOv8s, and SSD, across metrics such as precision, recall, and mAP@0.5. Particularly, it exhibits superior robustness and adaptability in detecting small objects such as safety helmets, reflective vests, and smoking behaviors.

Additionally, ablation experiments validate the independent contributions and synergistic effects of the GeoIoU loss function and the SEA module in improving model performance. GeoIoU significantly enhances the stability and accuracy of bounding box localization by introducing geometric features, while the SEA mechanism dynamically allocates feature weights, boosting the model’s feature representation capabilities in complex scenarios.

In summary, GeoIoU-SEA-YOLO not only provides an efficient and reliable solution for detecting unsafe behaviors on construction sites, but also offers new insights into addressing small object detection and multi-object interaction challenges in complex environments. Future research could focus on optimizing the lightweight design of the model to improve its deployment capabilities in resource-constrained environments and explore its generalizability and scalability across more complex scenarios and diverse tasks.

## Figures and Tables

**Table 1:** Hardware, software environment, and training strategy for model training.

Hardware	CPU	Intel(R)Core(TM)i7-13700KF (Intel, Santa Clara, CA, USA)
	RAM	32 GB
	GPU	NVIDIA GeForce RTX 4070 (NVIDIA, Santa Clara, CA, USA)
Software	CUDA	12.7
	Python	3.8.0
	Torch	2.3.1
Training strategy	Optimizer	Adam
	Batch size	16
	Learning rate	0.001

**Table 2:** Comparison of results between the proposed method and mainstream algorithms.

Model	AP (%)				Precision	Recall	mAP@0.5 (%)
	Hat	Person	Reflective	Smoking			
YOLOv3	0.811	0.846	0.860	0.837	0.784	0.742	0.839
YOLOv5s	0.854	0.882	0.891	0.870	0.822	0.765	0.874
YOLOv8s	0.881	0.910	0.922	0.903	0.851	0.787	0.904
SSD	0.826	0.861	0.873	0.850	0.803	0.750	0.852
GeoIou-SEA-YOLO	0.894	0.933	0.935	0.957	0.870	0.800	0.930

**Table 3:** Comparison of different model parameters and efficiency.

Model	Parameters (M)	FLOPs (G)	FPS
YOLOv8s	11.2	28.6	105
SSD	18.8	31.2	59
Faster R-CNN	41.0	160	18
GeoIou-SEA-YOLO	28.5	72.8	35

**Table 4:** Ablation study results on the independent impact of GeoIoU and SEA.

Model Configuration	GeoIoU	SEA	mAP@0.5	Precision	Recall
Baseline			0.822	0.765	0.874
YOLOv5s + GeoIoU	√		0.829	0.774	0.862
YOLOv5s + SEA		√	0.855	0.792	0.921
GeoIoU-SEA-YOLO	√	√	0.870	0.800	0.930

## Data Availability

The original contributions presented in the study are included in the article, further inquiries can be directed to the corresponding authors.
